# Expression of FSCN1 and FOXM1 are associated with poor prognosis of adrenocortical carcinoma patients

**DOI:** 10.1186/s12885-019-6389-3

**Published:** 2019-11-29

**Authors:** Jiayu Liang, Zhihong Liu, Xin Wei, Liang Zhou, Yongquan Tang, Chuan Zhou, Kan Wu, Fuxun Zhang, Fan Zhang, Yiping Lu, Yuchun Zhu

**Affiliations:** 1 Institute of Urology, Department of Urology, West China Hospital, Sichuan University, Chengdu, Sichuan China; 2 Department of Pediatric Surgery, West China Hospital, Sichuan University, Chengdu, Sichuan China

**Keywords:** Adrenocortical carcinoma, Prognosis, EMT, FSCN1, FOXM1

## Abstract

**Background:**

Adrenocortical carcinoma (ACC) is a rare malignant endocrine tumour. Due to a high tumour recurrence rate, the post-operative overall survival (OS) and disease-free survival (DFS) of ACCs is limited. Our research aims to identify the role of the epithelial-mesenchymal transition (EMT) related genes FSCN1 and FOXM1 in the tumour microenvironment and assess their prognostic value in ACCs.

**Methods:**

Clinical and specimen data from 130 adrenocortical carcinoma (ACC) patients was acquired from the Cancer Genome Atlas (TCGA) database (*n* = 79) and a West China Hospital (WCH) cohort (*n* = 51). In the WCH cohort, archived formalin-fixed paraffin embedded (FFPE) samples were collected for immunohistochemical analysis. The correlation between the EMT genes and the tumour microenvironment status was estimated based on the Tumour Immune Estimation Resource (TIMER) algorithm. Kaplan-Meier analysis, followed by univariate and multivariate regression analyses, were performed to identify the prognostic association of FSCN1 and FOXM1.

**Results:**

FSCN1 and FOXM1 were over-expressed in ACC tissue when compared with adrenocortical adenoma and normal adrenal tissue. Over-expression of FSCN1 or FOXM1 was associated with the tumour microenvironment and immune signatures in ACCs. Patients with higher expression of FSCN1 or FOXM1 were more likely to have worse prognoses. The prognostic effects were further verified in both early (stage I/II) and advanced (stage III/IV) ACCs. Furthermore, FSCN1 and FOXM1 appeared as independent prognostic factors in ACC.

**Conclusions:**

These results show that FSCN1 and FOXM1 are independent prognostic factors in ACCs and over-expression of FSCN1 or FOXM1 indicates a worse prognosis.

## Background

Adrenocortical carcinoma (ACC) is a rare endocrine malignancy with an estimated yearly incidence of 0.5–2.0 cases per million [1, 2]. Surgical resection is considered the first option for ACC; however, almost 50% of cases will develop recurrent or metastasis regardless of whether the initial complete resection was performed [3]. Identifying the prognostic factors of ACC remains a research focus. Currently known prognostic factors include tumour stage (according to the European Network for the study of Adrenal Tumours (ENSAT) classification) [4], resection (R) status [5, 6], Ki67 index [7] and some potential molecular biomarkers, such as vav guanine nucleotide exchange factor 2 (VAV2), topoisomerase II alpha (TOP2A) and a set of genes involved in DNA damage and regulation of cell-cycle pathways [8–11].

The tumourigenesis and development of ACC are poorly understood. Increasing evidence suggests that the epithelial-mesenchymal transition (EMT) may participate in adrenal tumourigenesis. According to those reports, EMT markers (E−/P−/N-cadherins, vimentin, fibronectin, MMP-2/− 9 and caveolin-1), and downstream transcriptional regulators (TWIST1, SIP1, Snail, ZEB-1/− 2, Slug) were all found to be dysregulated and associated with poor prognosis [12–14]. These results indicate a potential role of EMT in the development of ACC. In this research, our aim is to explore the clinicopathological and prognostic correlation of two EMT-related genes [15], FOXM1 and FSCN1.

Specifically, FOXM1 is recognized as a transcription factor of the Forkhead family. It is required for multiple processes in cancer development, and could also interact with other proteins to induce the oncogenic WNT and TGF-β signalling pathways [16], which are important in ACC development [17]. A study has found that the overexpression of FOXM1 in breast cancer, gastric cancer, hepatocellular carcinoma, pancreatic ductal adenocarcinoma, and non-small-cell lung cancer all predicted a worse survival [18]. FSCN1 encodes a member of the fascin family of actin-binding proteins and acts as a migration factor associated with EMT [19]. It is recognized to be associated with increased risk of mortality in breast, colorectal and oesophageal carcinomas [20]. More recently, the prognostic effect of FSCN1 was also observed in a European ACC cohort [21].

In the current study, we included The Cancer Genome Atlas (TCGA) cohort and a West China Hospital (WCH) cohort to explore the clinicopathological characteristics of FSCN1 and FXOM1 overexpressed ACCs. For the first time, the potential association between tumour microenvironment and FSCN1 and FOXM1 was identified. Based on the large number of ACC cases, we further compared the prognostic difference between patients with high- and low- FSCN1/FOXM1 expression.

## Methods

### Data collection and analysis

ACC clinical data and RNA-seq data from the TCGA project were analysed by GEPIA [22]. GEPIA used one-way ANOVA and the limma method in the differential expression analysis. The Kaplan-Meier curve method and Log-rank test were used in the survival analyses. This study meets the publication guidelines provided by TCGA (http:// cancergenome.nih.gov/publications/publicationguidelines). Microarray data (GSE12368) from the Gene Expression Omnibus database (GEO, http://www.ncbi.nlm.nih.gov/geo) were used to compare expression of candidate genes in ACC and benign adrenocortical adenoma (ACA) tissues [23]. Differentially expressed genes (DEGs) were analysed using GEO2R query and limma R packages from the Bioconductor project (http://www.bioconductor.org). Genes with an adjusted *P* value < 0.05 and a log2 fold change (logFC) > 1 were considered DEGs.

TIMER (https://cistrome.shinyapps.io/timer/) was used to estimate the potential association among candidate genes, immune cell infiltration and clinical parameters. The correlation between candidate genes with tumour microenvironment status was calculated based on six immune infiltrates (B cells, CD4+ T cells, CD8+ T cells, neutrophils, macrophages and dendritic cells) by Spearman’s correlation method, which was also validated using pathological estimations in the TIMER project [24]. Additionally, the candidate gene and immune signature correlation analysis was also validated in the GEPIA 2 platform (http://gepia2.cancer-pku.cn/#index) using the Pearson correlation coefficient method. The signatures were evaluated mainly based on specific markers in different types of immune cells. For example, the effector T cell signature depends on the expression level of CX3CR1, FGFBP2 and FCGR3A, and the effector memory T cell signature depends on PDCD1, DUSP4, GZMK, GZMA and IFNG.

### Patient cohort and ethical approval

Patients underwent resection for tumours at the West China Hospital and those that were pathologically confirmed as ACC from 2009 to 2016 were analysed. A total of 51 patients were enrolled in this study. The method and criteria of clinical record extraction and long-term follow-up were the same as our previous report [25]. Recurrent disease was diagnosed on the basis of clinical, radiographic, and laboratory evidence, including local recurrence, peritoneal carcinomatosis and distant metastases. Gender, age, grade, stage, treatment, R status, Ki67 index, and clinical follow-up data were updated. The corresponding formalin-fixed, paraffin-embedded (FFPE) tissues in our institutional biobank were retrospectively collected. This research was approved by the West China Hospital of Sichuan University Biomedical Research Ethics Committee following the ethical guidelines as required by the Declaration of Helsinki.

### Immunohistochemistry and image analysis

Serial FFPE tissue sections of 4-μm thickness were subjected to immunohistochemistry (IHC) analysis following standard protocols. Briefly, sections were deparaffinized in xylene and rehydrated through a graded ethanol series, then placed in 3% H2O2 for 15 min at room temperature. After the heat-mediated retrieval using sodium citrate or EDTA, slides were incubated with different primary antibodies: mouse anti-human Fascin (55 K-2) monoclonal antibody (#99978, Cell Signaling Technology, Danvers, MA, USA); rabbit anti-human FOXM1 monoclonal antibody (ab207298, Abcam, Cambridge, MA, USA), overnight at 4 °C. SignalStain® Boost IHC Detection Reagent (HRP, rabbit, CST) was applied for 30 min at room temperature according to the manufacturer’s instructions.

Immunostaining results were independently evaluated by two investigators blinded to the clinical data (F.Z. and C.Z.) and the inter-observer agreement was evaluated through the Cohen k coefficient value (0.86). A semi-quantitative H-score was calculated according to previous research [11] by multiplying the intensity score by the proportion score in which membrane and cytoplasmic staining intensity was evaluated with a score of 0 (negative), 1 (weak), 2 (moderate) or 3 (strong) and the proportion score was calculated as 0, 0.1, 0.5 or 1, respectively corresponding to 0%, 1–9%, 10–49% or > 50% of the positive tumour cells in each specimen. For the nuclear positive FOXM1, the proportion score was calculated as 1 or 2 according to the percentage of nuclear positive cells, as follows: 1, < 30% of cells positive; 2, ≥ 30% of cells positive. The cut-off value distinguishing high or low expression of candidate markers was H score ≥ 1 or < 1 (FSCN1) and H score > 1 or = 1 (FoxM1) in this research.

### Statistical analysis

Overall survival (OS) was defined as the time elapsed from primary resection of ACC to death due to any cause. Disease-free survival (DFS, also called relapse-free survival) was defined as the time elapsed from primary resection of ACC to the first recurrence (loco-regional or systemic). Clinicopathological categorical data were compared using Fisher’s exact tests. In the Cox regression analyses, gender, age, race, pathologic stage, CD8+ T cell signature, CD276, KLRB1, FSCN1 and FOXM1 were included in the multivariate Cox proportional hazards regression provided by TIMER. In the WCH cohort, gender, age, hormone secretion, laterality, tumour size, pathologic stage, symptoms, Ki-67 index, Surgery type, Fascin score and FoxM1 score were included in the univariate Cox proportional hazards regression. Next, variables with a *P* value < 0.10 were included in the multivariate Cox regression. Statistical analyses were performed using the R system (version 3.4.4) and GraphPad Prism version 6.02. A P value < 0.05 was considered statistically significant.

## Results

### FSCN1 and FOXM1 were overexpressed in ACC

**Fig. 1:**
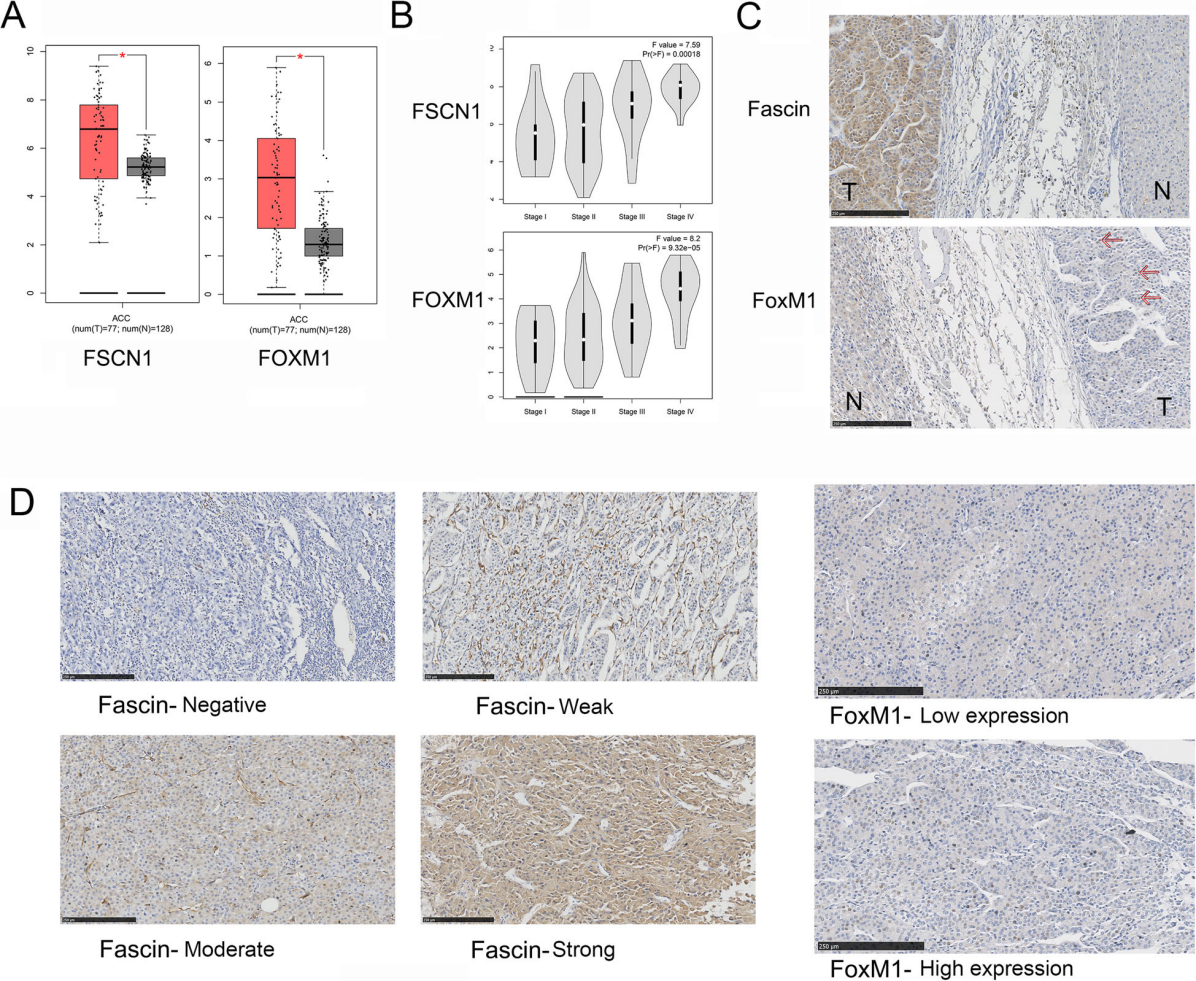
The expression level of FSCN1 and FOXM1 in ACC tissues. **a** The different expression level of FSCN1 and FOXM1 in ACC tissues and normal adrenal tissues (TCGA). **b** The average expression levels of FSCN1 and FOXM1 in I-IV stages (TCGA). **c** Immunohistochemical staining shows the higher expression of FSCN1 and FOXM1 in tumour tissues compared to adjacent normal tissues (WCH). **d** Immunohistochemical staining of Fascin and FoxM1 in ACC samples (WCH), with negative (low expression), weak (low expression), moderate (high expression) and strong (high expression) intensity. Scale bars: 250 μm

First, the mRNA expression levels of FSCN1 and FOXM1 in the TCGA ACC cohort were analysed. Both FSCN1 (logFC = 1.573, adjusted *P* < 0.001) and FOXM1 (logFC = 1.733, adjusted P < 0.001) were found to be over-expressed in ACC tissues (*n* = 77) compared to normal tissues (*n* = 128, Fig. 1a). Furthermore, patients at higher pathological stages were more likely to have a higher expression level of FSCN1 and FOXM1 (*P* < 0.001, Fig. 1b). The general characteristics of TCGA ACC patients were summarized in a previous report [21]. In addition, based on the microarray data (GSE12368) in ACCs (n = 12) and ACAs (*n* = 16), we also checked the different expression levels of FSCN1 and FOXM1. FSCN1 and FOXM1 were both overexpressed in ACCs (logFC = 1.28, adjusted *P* value = 0.00261 and logFC = 3.958, adjusted P value < 0.001, respectively).

### Expression and clinicopathological characteristics of Fascin (FSCN1) and FoxM1 (FOXM1) in the WCH cohort

Next, we validated the abnormal expression of Fascin (FSCN1) and FoxM1 (FOXM1) using ACC cases from the WCH cohort (*n* = 51) from 2009 to 2016 (Fig. 1b). The clinicopathological characteristics were analysed in Table 1. In the patient cohort, 4 (7.84%) were ENSAT stage I, 17 (33.3%) ENSAT stage II, 20 (39.22%) ENSAT stage III, and 10 (19.61%) ENSAT stage IV. The treatment and follow-up information were exhibited in Table 2. In this cohort, 34 (66.7%) patients underwent open adrenalectomy and 17 (33.3%) patients underwent laparoscopic adrenalectomy. The median age of WCH ACC patients was 44 (range from 3 to 79). Thirty-three (64.71%) patients died during follow-up, and 24 (47.06%) patients suffered from disease recurrence. Median overall follow-up was 723 (117–3210) days. The positive expression rate of Fascin and FoxM1 were 92.2% (47/51) and 78.4% (40/51), respectively. Representative sections and the different staining intensity grades of Fascin and FoxM1 were depicted in Fig. 1b and Fig. 1c. The correlation between the two markers and clinicopathological parameters were compared. Notably, patients at stage III/IV were more likely to have a high fascin expression status than those who were diagnosed at stage I/II (76.67% vs. 47.62%, *P* = 0.04). A high expression level of FoxM1 was more common in functional ACCs (82.61% vs. 32.14%, *P* < 0.001).

**Table 1:** clinicopathological characteristics of adrenocortical carcinomas in WCH cohort

Characteristics	Number (%)	FSCN1			FOXM1		
		High	Low	*p*	High	Low	*p*
Gender							
Female	33 (64.71)	23	8	0.06	17	16	0.57
Male	18 (35.29)	8	10		11	7	
Age							
< 65	44 (86.27)	30	14	0.23	25	19	0.69
≥ 65	7 (13.73)	3	4		3	4	
Hormone secretion							
No	28 (54.90)	17	11	0.57	9	19	0.001*
Yes	23 (45.10)	16	7		19	4	
Laterality							
Left	29 (56.86)	16	13	0.14	19	10	0.10
Right	22 (43.14)	17	5		9	13	
Tumor size (cm)							
< 7.5	27 (52.94)	20	7	0.16	15	12	1
≥ 7.5	24 (47.06)	13	11		13	11	
ENSAT tumor stage							
1	4 (7.84)	10	11	0.04*	10	11	0.41
2	17 (33.33)						
3	20 (39.22)	23	7		18	12	
4	10 (19.61)						
Ki67 index							
< 20%	30 (58.82)	16	14	0.07	15	15	0.57
≥ 20%	21 (41.17)	17	4		13	8	
Symptoms at diagnosis							
No	15 (29.41)	7	8	0.11	6	9	0.22
Yes	36 (70.59)	26	10		22	14

**Table 2:** Treatment and follow-up data in WCH cohort

Characteristics	Number (%)
Surgery type	
Open	34 (66.7)
laparoscopic	17 (33.3)
Re-operation on recurrence cases	9 (17.6)
Mitotane	4 (7.8)
Other (radiation, radiofrequency ablation, other chemotherapies)	4 (7.8)
Margin status (R1/2/X)	13 (25.5)
Duration of follow up – days (median, range)	723 (117–3210)
Survival status (during follow up)	
Recurrences	24 (47.06)
Deaths	33 (64.71)

### Overexpression of FSCN1 and FOXM1 were associated with immune status of ACC

**Fig. 2:**
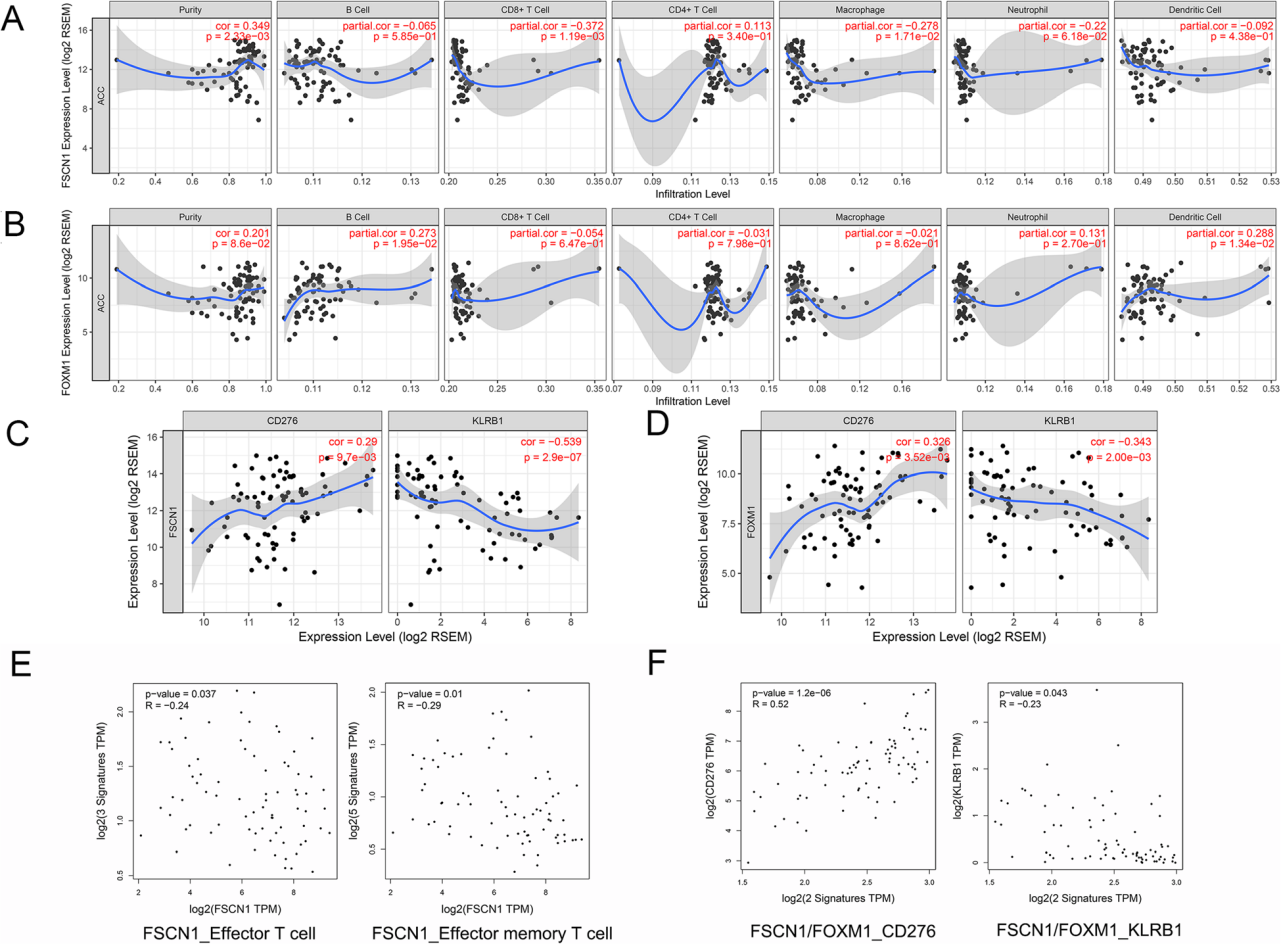
The association between FSCN1/FOXM1 and immune signature (TIMER and GEPIA 2 analyses). **a** The correlation between immune cell infiltration and the expression levels of FSCN1. **b** The correlation between immune cell infiltration and the expression levels of FOXM1. **c** Co-expression of CD276/KLRB1 and FSCN1 in ACC. **d** Co-expression of CD276/KLRB1 and FOXM1 in ACC. **e** The correlation between FSCN1 signature and effector T cell and effector memory T cell signatures. **f** The associations between merged FSCN1/FOXM1 signature and immune related genes CD276 and KLRB1, respectively

To explore their potential correlation with the immune environment in ACC, the relationships between FOXM1/FSCN1 and tumour immunity were evaluated based on the TCGA-ACC cohort in TIMER. FSCN1 expression was found to be positively correlated with ACC tumour purity (r = 0.349, *p* = 2.33e− 03), and negatively correlated with the infiltration signature of CD8+ T cells (r = − 0.372, *p* = 1.19e – 03, Fig. 2a), while FOXM1 showed a weak correlation with B cell and dendritic cell signatures (Fig. 2b). Given that an association between FOXM1/FSCN1 and immune signature was observed in our previous study [26], we further explored the potential links between FSCN1/FOXM1 and several immune markers, such as CD276 and the innate immune marker, CD161 (KLRB1) [26–28]. Notably, Both FSCN1 and FOXM1 were found to be negatively correlated with innate immune signature, KLRB1(r = − 0.539, *P* value = 2.9e-07, and r = − 0.343, P value = 2.0e-03, respectively), and positively correlated with CD276 (r = 0.29, P value = 9.7e-03 and r = 0.326, P value = 3.52e-03, respectively, Fig. 2c and d). To validate the immune correlation, we further performed GEPIA 2 analysis. A negative correlation was observed between FSCN1 signature and effector T cell signature and effector memory T cell signature (Fig. 2e). We also merged the FSCN1 and FOXM1 signature and verified its correlation with CD276 and KLRB1 (Fig. 2f). As expected, the CD276 signature was significantly correlated with the FSCN1/FOXM1 signature (r = 0.52, P value = 1.2e-06). The KLRB1 signature was negatively associated with the FSCN1/FOXM1 signature (r = − 0.23, P value = 0.043). These results indicated that the FSCN1- and FOXM1-signatures might be involved in immune activities in the ACC microenvironment.

### Overexpression of FSCN1 and FOXM1 were correlated with poor prognosis of ACC patients

**Fig. 3:**
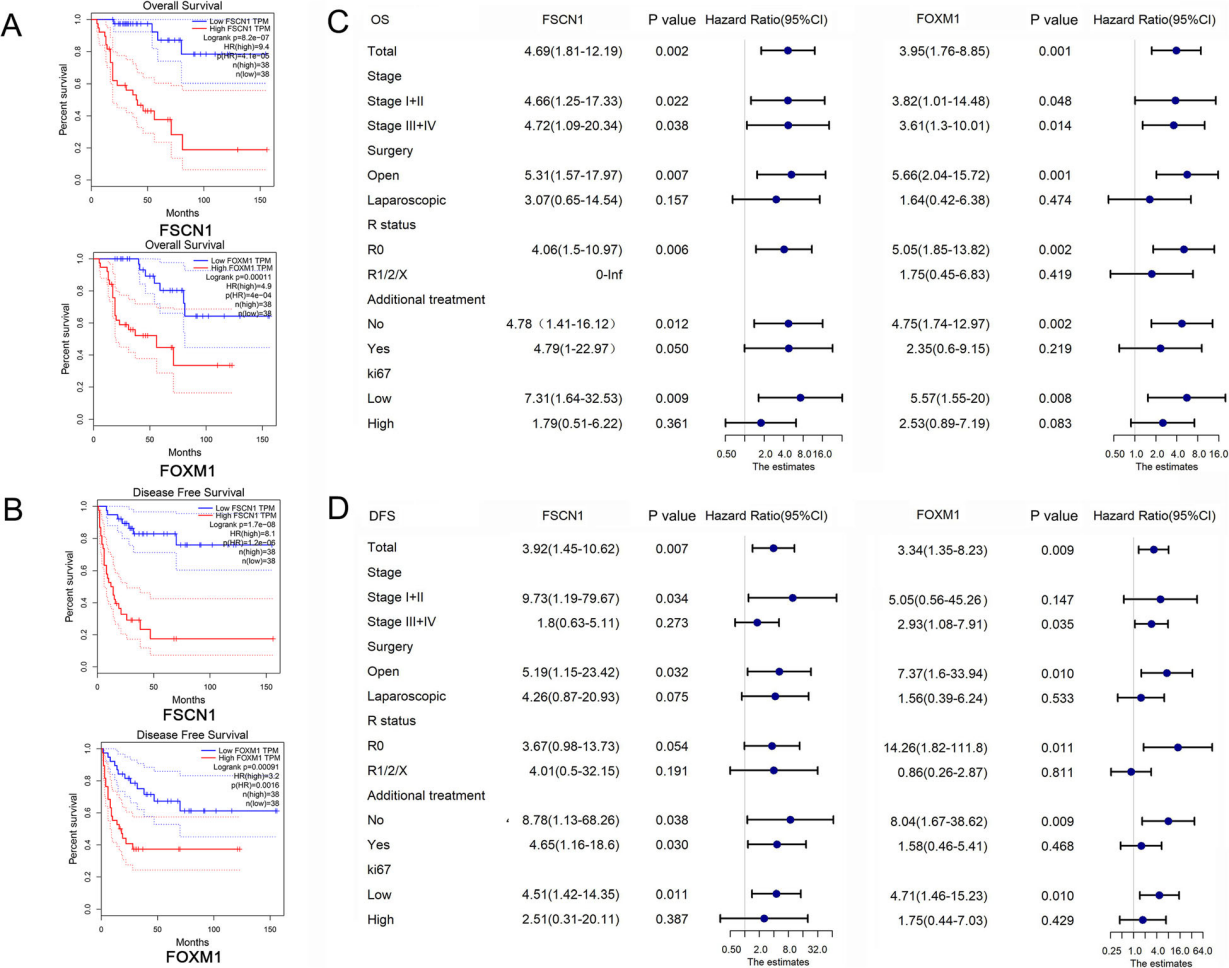
The association between FSCN1/FOXM1 and prognosis of ACC. **A** Overall survival for FSCN1 and FOXM1 expression (TCGA, *n* = 76). **B** Disease-free survival for FSCN1 and FOXM1 expression (TCGA, n = 76). **C** Univariate prognostic effects of FSCN1 and FOXM1 in subgroup analysis for OS (WCH, *n* = 51). **D** Univariate prognostic effects of FSCN1 and FOXM1 in subgroup analysis for DFS (WCH, n = 51)

In the TCGA ACC cohort, patients with a high expression level of FOXM1 and FSCN1 had worse overall survival (OS, *n* = 76, HR = 4.9, *P* = 4e-04 and HR = 9.4, *P* = 4.1e-05, respectively, Fig. 3a) and disease-free survival (DFS, n = 76, HR = 3.2, *P* = 0.0016 and HR = 8.1, *P* = 1.2e-06, respectively, Fig. 3b). Based on the multivariable Cox proportional hazard model in TIMER, we explored the clinical relevance of FSCN1 and FOXM1 with the flexibility to correct for multiple covariates including gender, age, race, pathologic stage, CD8+ T cell signature, CD276 and KLRB1 (Table 3). The results suggested that FSCN1 and FOXM1 had independent prognostic effects on ACC patients (*P* < 0.001 and P = 0.001, respectively).

**Table 3:** Multivariate analyses of parameters associated with OS in TCGA cohort

	Multivariate analysis		
Variables	Hazard Ratio	95% CI	*p*
Age	1.026	0.991–1.063	0.153
Gender (male)	2.128	0.647–7.001	0.214
Race (Black)	0.616	0.000-Inf	NA
Stage I	–	–	–
Stage II	5.819	0.397–85.33	0.199
Stage III	4.464	0.354–56.72	0.247
Stage IV	3.720	0.294–4.699	0.310
CD8^+^ T cell	1307	0–1.71e+ 11	0.452
FSCN1	2.314	1.397–3.831	0.001*
FOXM1	2.821	1.539–5.173	0.001*
CD276	1.002	0.522–1.922	0.996
KLRB1	0.824	0.568–1.194	0.307

Moreover, the prognostic significance of Fascin (FSCN1) and FoxM1 (FOXM1) were also observed in the WCH cohort. High- and low- expression of two proteins were classified according to the expression score. As a result, high Fascin expression and high FoxM1 expression in the tumour tissues were both remarkably correlated with worse OS (HR = 4.69, *P* = 0.002 and HR = 3.95, P = 0.001, respectively) and DFS (HR = 3.92, *P* = 0.007 and HR = 3.34, *P* = 0.009, respectively). In the multivariate Cox model, Fascin score (HR = 2.86, 95%CI: 1.06–7.7, *P* = 0.038), FoxM1 score (HR = 3.17, 95%CI: 1.37–7.33, P = 0.007) and Ki67-index (HR = 2.39, 95%CI: 1.15–4.99, *P* = 0.02) were verified as independent risk factors for OS. Fascin score (HR = 2.98, 95%CI: 1.02–8.7, *P* = 0.046), FoxM1 score (HR = 2.98, 95%CI: 1.14–7.8, *P* = 0.026) and symptoms (HR = 4.10, 95%CI: 1.11–15.19, *P* = 0.035) were verified as independent risk factors for DFS (Table 4).

**Table 4:** Univariate and multivariate COX analyses of parameters associated with OS and DFS in WCH cohort

	Univariate analysis			Multivariate analysis		
Variables	Hazard Ratio	95% CI	P Value.	Hazard Ratio	95% CI	P Value
OS						
Gender (Male)	0.93	0.45–1.91	0.833	–	–	–
Age (≥65)	1.61	0.66–3.92	0.294	–	–	–
Hormone secretion (Yes)	0.78	0.39–1.58	0.494	–	–	–
Laterality (Right)	0.85	0.42–1.73	0.663	–	–	–
Tumor Size (≥7.5)	0.71	0.35–1.41	0.322	–	–	–
Stage (III + IV)	1.48	0.73–3.03	0.278	–	–	–
Ki67 index (High)	2.86	1.41–5.81	0.004	2.39	1.15–4.99	0.02
Symptoms (Yes)	3.45	1.32–9.05	0.012	2.22	0.82–5.96	0.115
Fascin	4.69	1.81–12.19	0.002	2.86	1.06–7.7	0.038
FoxM1	3.95	1.76–8.85	0.001	3.17	1.37–7.33	0.007
Surgery type	0.88	0.42–1.88	0.745			
DFS						
Gender (Male)	0.65	0.27–1.59	0.351	–	–	–
Age (≥65)	1.12	0.33–3.8	0.859	–	–	–
Hormone secretion (Yes)	1.48	0.65–3.36	0.350	–	–	–
Laterality (Right)	1.38	0.61–3.11	0.439	–	–	–
Tumor Size (≥7.5)	0.81	0.36–1.85	0.624	–	–	–
Stage (III + IV)	3.58	1.31–9.79	0.013	2.81	1–7.95	0.051
Ki67 index (High)	1.45	0.61–3.42	0.397	–	–	–
Symptoms (Yes)	5.14	1.49–17.64	0.009	4.10	1.11–15.19	0.035
Fascin	3.92	1.45–10.62	0.007	2.98	1.02–8.7	0.046
FoxM1	3.34	1.35–8.23	0.009	2.98	1.14–7.8	0.026
Surgery type	1.83	0.79–4.26	0.160	–	–	–

In addition, the prognostic difference in the Fascin and FoxM1 high−/low- groups stratified by stage, treatment, margin status (R), additional adjuvant therapy and Ki67 index were also evaluated using the univariate Cox proportional hazards regression method. Patients were divided into these subgroups to check if the Fascin/FoxM1-related prognostic effects could be observed. Accordingly, both Fascin and FoxM1 were found to correlate with OS in early (I/II) or late (III/IV) stage ACCs (Fig. 3c). This OS-correlation relationship was also shown in the open surgery subgroup, R0 subgroup, no-additional adjuvant therapy treatment subgroup and low-Ki67 subgroup (Fig. 3c). On the other hand, the DFS-correlation of Fascin and FoxM1 were respectively observed in early stage and late stage ACCs. The same effects were also found in the open surgery subgroup, no-additional adjuvant therapy treatment subgroup and low-Ki67 subgroup (Fig. 3d).

## Discussion

As a type of aggressive tumour, the factors involved in tumour progression and metastasis of ACC remains unclear. In this study, we assessed the clinical significance of FSCN1 and FOXM1 based on RNA expression data from the TCGA ACC cohort and IHC staining results from a West China Hospital cohort. Both EMT-related genes were found to be overexpressed and correlated with immune signatures and prognosis of ACC patients. These findings increased our understanding of the potential role of a metastatic signature in ACC.

As described recently in the Florence ACC series (*n* = 37), Kaplan-Meier analysis of immunohistochemical expression also showed a significant correlation between Fascin-1 and prognosis of ACC [21]. These consistent findings from different countries and races further strengthen our results and confirmed the potential of FSCN1 as a prognostic marker in ACC. Additionally, we have shown that although a significant correlation between FSCN1/FOXM1 and advanced clinical stage was found, they were considered as independent prognostic indicators along with the well-recognized Ki-67 index in the overall survival analysis.

Clinically, multiple parameters were reported to be correlated with overall survival of ACC patients, including age, hormone secretion, Weiss score, ki67 index and the resection (R) status [29–34]. R. Libé et al. analysed the advanced ACC in ENSAT dataset and found that GRAS parameters (Grade, R status, Age and Symptoms) successfully stratified the different prognosis of patients [35]. Adjuvant mitotane significantly decreased the recurrence rate and mortality after resection of ACC in patients without distant metastasis [36]. However, due to the small proportion of ACC patients receiving mitotane or other adjuvant therapy treatment in our cohort (*n* = 8), the prognostic influence of FSCN1 and FOXM1 in the subgroup for adjuvant therapy were not successfully observed.

The prognostic influence of FSCN1 and FOXM1 were also assessed in subgroups for different treatments, margin status and ki-67 status. For patients who underwent open surgery, had negative margin status or a low ki-67 index, higher expression of FSCN1 or FOXM1 could be regarded as a potential risk for poor prognosis. In addition to those well-recognized clinicopathological parameters, we further identified that these two EMT-related genes may be used as biomarkers for low-risk ACCs in a traditional sense.

During the initial process of EMT-related metastasis and invasion, the changes that suppress anti-tumour immunity in the tumour microenvironment are occurring in parallel [37]. Chen L et al. reported that the miR-200/ZEB1 axis could regulate the EMT signature on tumour cells and simultaneously target the PD-L1, leading to CD8+ T cell immunosuppression and metastasis [38]. Here, we observed that as one of the most promising T cell target antigens, FSCN1 is also thought to be positively correlated with T cell response [39]. Thus, the newfound negative correlation between FSCN1 and CD8+ T cells further indicates the existence of a potential immunosuppressive signal.

The limitations of this study include that, due to the limited frozen ACC samples available, we did not perform additional full-quantitative experiments to validate the expression of FSCN1 and FOXM1 mRNA in the WCH cohort. The underlying mechanism of the association between FSCN1/FOXM1 and an immune signature is unclear and requires further investigation.

## Conclusions

Overexpression of FSCN1 and FOXM1 were correlated with immune status in the ACC microenvironment. Both EMT-related genes were regarded as independent prognostic factors in ACC.

## Data Availability

The datasets used and/or analysed during the current study are available from the corresponding author on reasonable request.

## Abbreviations

ACC: Adrenocortical carcinoma
EMT: epithelial-mesenchymal transition
IHC: immunohistochemistry
